# Polysaccharide biosynthesis-related genes explain phenotype-genotype correlation of *Microcystis* colonies in Meiliang Bay of Lake Taihu, China

**DOI:** 10.1038/srep35551

**Published:** 2016-10-18

**Authors:** Shutu Xu, Qianqian Sun, Xiaohua Zhou, Xiao Tan, Man Xiao, Wei Zhu, Ming Li

**Affiliations:** 1 College of Agronomy, Northwest A & F University, Yangling 712100, PR China; 2 College of Resources and Environment, Northwest A & F University, Yangling 712100, PR China; 3 College of Environment, Hohai University, Nanjing 210098, PR China; 4 Australian Rivers Institute, Griffith University, Nathan, Qld 4111, Australia; 5 Key Laboratory of Plant Nutrition and the Agri-environment in Northwest China, Ministry of Agriculture, PR China

## Abstract

The 16S rDNA, 16S-23S rDNA-ITS, *cpc*BA-IGS, *mcy* gene and several polysaccharide biosynthesis-related genes (epsL and *TagH*) were analyzed along with the identification of the morphology of *Microcystis* colonies collected in Lake Taihu in 2014. *M. wesenbergii* colonies could be distinguished directly from other colonies using *espL*. *TagH* divided all of the samples into two clusters but failed to distinguish different phenotypes. Our results indicated that neither morphology nor molecular tools including 16S rDNA, 16S-23S ITS and *cpc*BA-IGS could distinguish toxic and non-toxic species among the identified *Microcystis* species. No obvious relationship was detected between the phenotypes of *Microcystis* and their genotypes using 16S, 16S-23S and *cpc*BA-IGS, but polysaccharide biosynthesis-related genes may distinguish the *Microcystis* phenotypes. Furthermore, the sequences of the polysaccharide biosynthesis-related genes (*espL* and *TagH*) extracted from *Microcystis* scums collected throughout 2015 was analyzed. Samples dominated by *M. ichthyoblabe* (60–100%) and *M. wesenbergii* (60–100%) were divided into different clade by both *espL* and *TagH*, respectively. Therefore, it was confirmed that *M. wesenbergii* and *M. ichthyoblabe* could be distinguished by the polysaccharide biosynthesis-related genes (*espL* and *TagH*). This study is of great significance in filling the gap between classification of molecular biology and the morphological taxonomy of *Microcystis*.

*Microcystis* spp. is a common genus of bloom-forming cyanobacteria, which generates *Microcystis* blooms worldwide^1^. *Microcystis* blooms is one of the serious harmful algae blooms because many *Microcystis* species produce microcystins having high toxicity^2^. These blooms also cause fish mortality due to depletion of oxygen^3^ and loss of biodiversity and affect the cycles of biogenic elements in freshwater ecosystems^1^,^4^. Thus, an insight into the distribution, succession and diversity of *Microcystis* species is important to understand the life-cycle of *Microcystis* as well as ecology of *Microcystis* blooms.

During the past decades, many studies have been carried out to investigate the processes of *Microcystis* bloom formation^5^,^6^. Multiple *Microcystis* species have been recorded according to their morphological characteristics, especially their colonial morphology^7^. The life cycle^8^, spatial distribution^9^, seasonal succession^10^ and physiology of *Microcystis*^11^ has been well studied based on this morphological taxonomy. In addition, the competition between *Microcystis* spp. and other algae and also the competition among different *Microcystis* species have been investigated to reveal the ecology of *Microcystis* bloom formation^12^,^13^.

Recently, *Microcystis* has been well documented having high phenotypic plasticity^14^,^15^. Otsuka *et al*.^16^ demonstrated that the colonial morphology of *Microcystis* in culture could change from time to time. Sun *et al*.^17^ indicated that colonies with colonial morphology of *M. aeruginosa* under culture conditions could change their morphology to that of a typical *M. novacekii*. Li *et al*.^18^ illustrated that solubilization of mucilage could induce changes in colonial morphology and the authors suggested that seasonal succession of *Microcystis* species was due to morphological changes. Therefore, the taxonomy of this genus should be re-evaluated via molecular genetic analyses.

The phenotype-genotype correlation of *Microcystis* is helpful in filling the gap between classification of molecular biology and the morphological taxonomy of *Microcystis*. The phylogenetic analysis based on 16S rDNA was considered as one of the most reliable criteria for determining relationships among organisms with close relation^19^. However, the similarity of colonies in different morphology was high as measured by 16S rDNA sequencing^20^,^21^, and thus the unification of five species of *Microcystis* has been proposed^22^. In addition, the events of horizontal gene transfer would cause flexibility of several informative genes including 16S rDNA of *Microcystis*^23^. A more reliable gene sequence should be explored to analyze the phenotype-genotype correlation of *Microcystis*. Otten and Paerl^24^ indicated that *M. wesenbergii* could be identified from four different *Microcystis* morphospecies using 16S-23S rDNA-ITS sequences, but the other four morphospecies could not. Tan *et al*.^25^ indicated *cpc*BA-IGS could be used as an effective tool to identify *M. wesenbergii*. Several polysaccharide biosynthesis-related genes were also found to identify morphospecies of *Microcystis*^26^. Thus, these genes were hypothesized to be significantly related to *Microcystis* colonial morphology, and this hypothesis has been preliminarily verified by Xu *et al*.^27^.

In addition, microcystin-producing genes were also postulated to divide *Microcystis* into toxic species and non-toxic species^28^. The morphospecies was considered to relate to the toxicity of *Microcystis*. Generally, *M. ichthyoblabe* was considered as non-toxic species^29^, while *M. aeruginosa* and *M. wesenbergii* as toxic species^30^,^31^,^32^. The microcystin synthetase (*mcy*) gene cluster in different *Microcystis* morphospecies was thus analyzed to reveal the phenotype-genotype correlation of *Microcystis* colonies^33^. However, it was still poorly understood whether there was a relationship between the phenotype and microcystin-producing genes.

The current study aimed to gain insight into the phenotype-genotype correlation of *Microcystis*. The 16S rDNA, 16S-23S rDNA-ITS, *cpc*BA-IGS, *mcy* gene (*mcy*B)^34^ and several polysaccharide biosynthesis-related genes were analyzed along with the identification of the morphology of *Microcystis* colonies collected in the field. This study also attempted to resolve that polysaccharide biosynthesis-related genes might distinguish the *Microcystis* morphospecies as EPS played great roles in colony formation and morphological changes of *Microcystis*^18^,^35^.

## Materials and Methods

### Experimental design

This study has two parts. (I) Seeking novel functional gene which may distinguish the *Microcystis* morphospecies. Individual *Microcystis* colonies were isolated from natural samples and then axenically cultured for PCR amplification and sequencing. Afterwards, phenotype-genotype correlation of *Microcystis* colonies was investigated and the function gene was identified. (II) Confirming the functional gene. *Microcystis* “scum” at different seasons were collected and divided into varying classes consisting of various *Microcystis* morphospecies according to colony size. The functional genes of the subsamples were then analyzed to confirm that this gene succeed in distinguishing the *Microcystis* morphospecies.

### Sample collections

Algal samples for colony isolation and culture in part I were collected during a *Microcystis* bloom in Meiliang Bay in northern Lake Taihu (China) on 15 August and 1 November 2014. Lake Taihu was selected in the current study because *Microcystis* spp. is the dominant species at most of the time and heavy *Microcystis* blooms occurs frequently^10^. In addition, the colony morphology and phylogenetic inference of *Microcystis* species has been well investigated in this lake^8^,^24^,^36^, which could be referred to. The water samples containing abundant *Microcystis* colonies were collected directly from the lake surface (30 cm depth) and were transferred into plastic bottles with a capacity of 5 L. The samples were then stored in a cold closet and transported to the laboratory as soon as possible for culture. Algal samples for confirming the functional gene in part II were collected on 4 June, 16 July, 17 August, 29 September, 15 October and 15 November, 2015, respectively.

### Microcystis colony separation

Water samples for part I were diluted with BG-11 culture medium until a single *Microcystis* colony could be separated by a pipette. The separated colony was examined under a microscope (×100), and the colonial morphology was recorded. *M. aeruginosa* and *M. wesenbergii* were found in the sample collected on 15 August. *M. ichthyoblabe* was found in the sample collected on 1 November. Five colonies of each morphology were separated for culture. *M. ichthyoblabe* colonies were named *M. ichthyoblabe* colonies TH11, TH12, TH13, TH14 and TH15. *M. aeruginosa* colonies were named *M. aeruginosa* colonies TH21, TH22, TH23, TH24 and TH25. *M. wesenbergii* colonies were named *M. wesenbergii* colonies TH31, TH32, TH33, TH34 and TH35.

### Single colony culture

Each colony was washed with BG-11 medium three times. Then, the colonies were cultured in 10 mL of BG-11 medium in glass tubes at 25 °C under a 12 h:12 h light-dark cycle with a light density of approximately 45 μmol m^−2^ s^−1^. After one month of culture, the *M. ichthyoblabe* colonies TH11, TH12, TH13, TH14, TH15, the *M. aeruginosa* colonies TH21 and TH22 and the *M. wesenbergii* colonies TH31 and TH32 grew well but the others died. The DNA of the growing *Microcystis* was extracted.

### DNA extraction

The DNA extraction method was referred to Sun *et al*.^17^. *Microcystis* pellets were dispersed into 0.8 mL extraction buffer (1.5 M NaCl, 1% CTAB, 100 mM Tris-HCl, 100 mM Na_2_EDTA, 100 mM Na_3_PO_3_, pH 0.8) and 20 μL of proteinase K (30 mg mL^−1^). Afterwards, they were incubated at 37 °C for 30 min and then, 0.48 mL of 20% SDS was added to each sample, incubating at 65 °C for 1 h. The samples were extracted using phenol-chloroform-isoamyl (25:24:1) and chloroform-isoamyl (24:1) successively. Centrifuged at 8000 × g for 5 min, the supernatant was transferred to new tubes. Thereafter, 0.6 mL pure isopropyl alcohol was injected to purify the DNA sample. After 20-min centrifugation at 16000 × g, 70% ethanol was used to rinse the DNA sample. Each DNA sample was dried and dissolved in 100 μL of Tris-EDTA (10 mM Tris and l mM EDTA, pH 8.0). Finally, the DNA sample was analyzed using a Nanodrop-2000.

### PCR amplification and sequencing

Seven pairs of primers targeting the 16S rRNA, 16S-23S ITS(A)/(S), *cpc*BA-IGS, *mcy*B, *Tag*H and *eps*L genes were used for the amplification and sequencing of all of the samples (see Table 1). A total volume of 50 μL containing 25 μL of 2 × PCR mixture buffer with tag enzyme (Bioteke, Beijing, China), 1.2 μL of each primer (10 μM), 2 μL DNA (10–20 ng μL^−1^) and 21.8 μL ddH_2_O was used for the PCR amplifications. The PCR amplification was run with an initial denaturation of the DNA at 94 °C for 5 min, followed by 34 cycles of 50 s at 94 °C, 50 s at 42 °C (*mcy*B) or 30 s at 50 °C (16S, 16S-23S) or 30 s at 52 °C (*cpc*BA-IGS) or 30 s at 55 °C (*Tag*H, *eps*L), and 1 min at 72 °C. The reaction was completed after 10 min at 72 °C. The detection and the size of the amplicons were determined by agarose (1.0%) gel electrophoresis compared with a DL2000 DNA Marker (Tiangen, Beijing, China). The amplicons with the correct length were used for sequencing by the Tianyihuiyuan biotechnology company (except *mcy*B gene).

### Treatment of samples for part II

**Figure 1:**
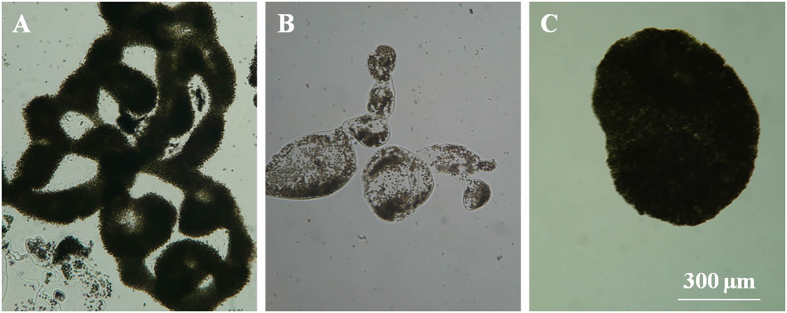
Micrographs of *Microcystis* species collected in Lake Taihu. (**A**) *M. aeruginosa*; (**B**) *M. wesenbergii*; (**C**) *M. ichthyoblabe*.

The sample for part II was poured gently through sieves (divided into four classes: >500 μm, 300–500 μm, 150–300 μm and 75–150 μm). Each class was re-suspended in BG-11 medium. For each subsample from sieving, the photomicrographs were taken using an Olympus C-5050 digital camera coupled with an optical microscope (Olympus CX31). The length and width of *Microcystis* colonies was analyzed using the UTHSCSA ImageTool (v3.00, University of Texas Health Science Center, San Antonio, TX, USA). The biovolume of *Microcystis* colony was calculated as volume = π/6 (length × width)^3/2^ as it is hard to measure the thickness of colonies. A total of 300 colonies were analyzed in each sample. Afterwards, the percentage of different *Microcystis* morphospecies in the total *Microcystis* biovolume of each subsample was calculated. *Microcystis* morphospecies was identified according to Yu *et al*.^7^. In the current study, *M. ichthyoblabe*, *M. aeruginosa* and *M. wesenbergii* was identified as in Fig. 1 and other *Microcystis* colonies were defined as unidentified *Microcystis*.

For each subsample, DNA for PCR templates was extracted. Only *epsL* and *TagH* were used for amplification and sequencing according to the results of part I. All the procedure and method was as same as those described for part I.

### Data analysis

Alignment for all of the sequences was determined by Muscle and edited by software Bioedit^37^. Some related sequences in the NCBI database were also used for alignment. MEGA5 was used to construct neighbor-joining tree of phylogeny analysis^38^, with bootstrap for 1000 replications, Maximum Composite Likelihood, and d: Transitions + Transversions.

## Results and Discussion

### Relationship between species and toxicity

**Figure 2:**
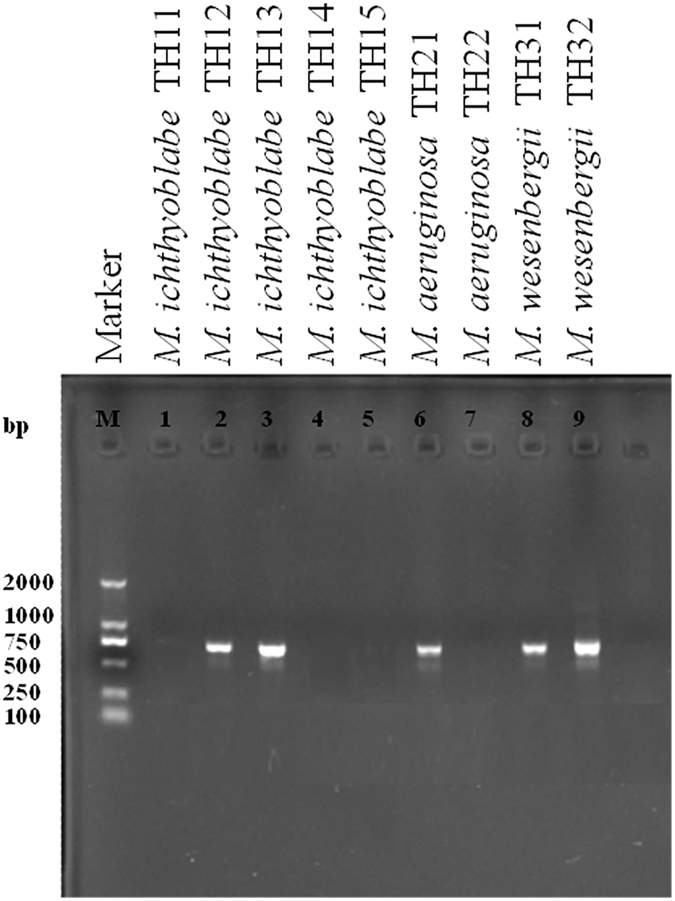
Electropherogram of the PCR products with the primer of *mcy*B.

Figure 2 shows an electropherogram of the PCR products with the primer of *mcy*B. Our results showed that one *M. aeruginosa* colony contained *mcy*B but the other did not. Two out of five *M. ichthyoblabe* colonies contained *mcy*B in this study. Mazur-Marzec *et al*.^39^ showed similar results in the Vistula Lagoon (southern Baltic Sea). However, *M. aeruginosa* colonies are generally considered as toxic species^30^,^40^. *M. ichthyoblabe* has never been reported to produce microcystins^29^,^41^,^42^. *M. wesenbergii* was classified as a non-toxic species^31^, but our results showed that both two *M. wesenbergii* colonies contained *mcy*B. Nevertheless, some investigations^32^,^42^ also illustrated that *M. wesenbergii* is toxic. All of the conflicting conclusions above indicated that there is not an exact relationship between the phenotype and microcystin-producing genes.

Yoshida *et al*.^32^ divided 47 strains of *Microcystis* into three clusters based on the sequences of 16S-23S rDNA-ITS. Their results showed that the first cluster contained both non-toxic and toxic strains, the second only had toxic ones, and the last only had non-toxic strains. This result implied that the 16S-23S gene may distinguish the toxic and non-toxic *Microcystis* species, which was also reported by Janse *et al*.^43^. On the contrary, our results demonstrated that the 16S-23S gene sequences failed to distinguish nine strains with different phenotypes, four of which possessed the *mcy*B gene. This result suggested that 16S-23S rDNA-ITS gene failed to distinguish toxic and non-toxic strains. Yoshida *et al*.^44^ suggested that 16S rDNA could used to identify toxic and non-toxic *Microcystis* species in some bloom stages. However, our results did not reach a similar conclusion. Therefore, the *Microcystis* species identified by morphology or molecular tools (16S rDNA, 16S-23S ITS and *cpc*BA-IGS) could not be used to distinguish toxic and non-toxic species.

### Phylogenetic trees based on 16S, 16S-23S and cpcBA-IGS

**Figure 3:**
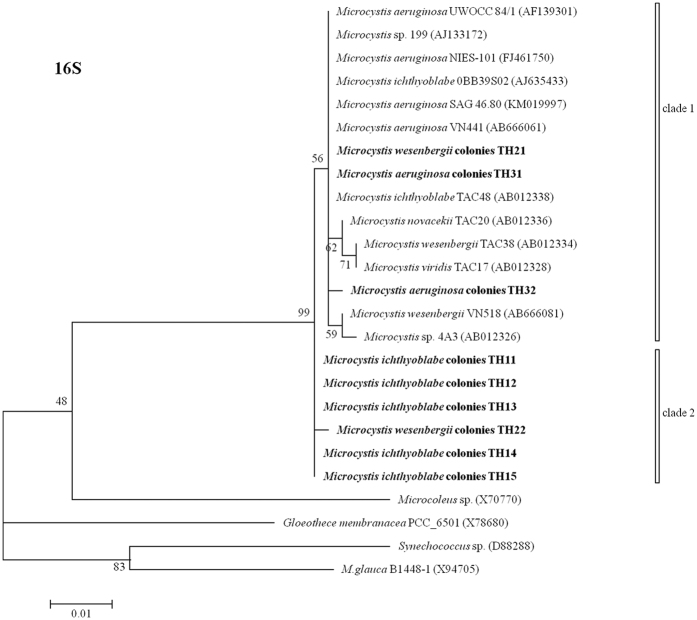
Phylogenetic tree based on the analysis of the 16S gene sequences.

**Figure 4:**
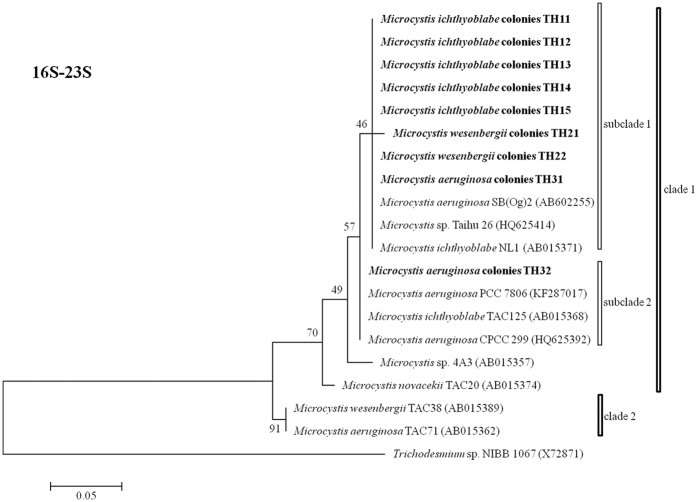
Phylogenetic tree based on the analysis of the 16S-23S gene sequences.

**Figure 5:**
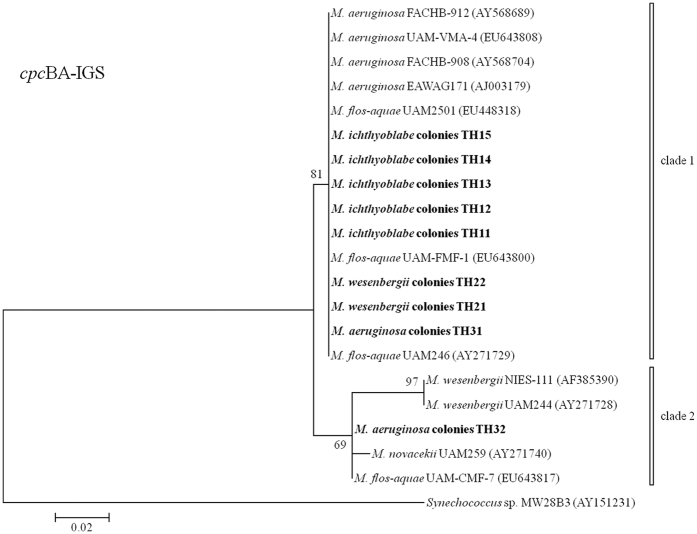
Phylogenetic tree based on the analysis of the *cpc*BA-IGS gene sequences.

The phylogenetic trees referring to 16S, 16S-23S and *cpc*BA-IGS are illustrated in Figs 3, 4 and 5, respectively. The 16S sequences divided all of the samples into two clusters. All of the *M. ichthyoblabe* colonies were in the same clade, but this clade also included *M. wesenbergii* colony (TH22). Both of the *M. aeruginosa* colonies and *M. wesenbergii* colonies were found in clade 1. However, these colonies had high homozygosity in 16S with *M. ichthyoblabe* 0BB39S02 (AJ635433), *Microcystis novacekii* TAC20 (AB012336) and *Microcystis viridis* TAC17 (AB012328). 16S rDNA sequences could not be used to distinguish different phenotypes of *Microcystis*^20^. Lepère *et al*.^21^ also reported that the 16S rDNA sequences of six *Microcystis* strains assigned to four different morphospecies based on colonial morphology were similar.

Sanchis *et al*.^45^ used both the 16–23S rDNA ITS and the cpcBA-IGS sequences to identify *Microcystis*. Their results suggested that *M. novacekii* could be distinguished from *M. wesenbergii*, but there was a close relationship between *M. novacekii* and *M. aeruginosa*. Otten and Paerl^24^ also indicated that *M. wesenbergii* could be identified within four different *Microcystis* morphospecies based on the 16S-23S rDNA-ITS sequences. Similarly, Yoshida *et al*.^32^ found that *M. aeruginosa* could be distinguished from *M. wesenbergii* and *M. viridis* by the 16S-23S rDNA-ITS sequences. Do Carmo Bittencourt-Oliveira *et al*.^46^ successfully distinguished the *M. aeruginosa* morphospecies from the morphospecies of *M. wesenbergii* and *M. viridis* based on the DNA sequences of *cpc*BA-ITS.

All the above studies considered that *M. wesenbergii* could be distinguished using the 16–23S rDNA ITS and the cpcBA-IGS sequences. Conversely, in the current study, the sequences displayed high homozygosity for each 16S-23S and *cpc*BA-IGS in all of the samples except for the *M. aeruginosa* colony, TH32 (Figs 4 and 5). Similarly, the phylogenic tree for the 63 *Microcystis* strains in China based on the *cpc*BA-IGS gene sequences showed that this gene did not always succeed in identifying different morphospecies^47^. These occasional failures may be resulted from genetic variations among the strains of *Microcystis*^48^. One *Microcystis* genotype was reported to have more than one phenotype^29^,^49^. In East Africa, 24 isolated strains of *M. aeruginosa* could be separated into 10 genotypes based on the DNA sequences of the PC-IGS and ITS1 rDNA regions^50^. Thus, there was no obvious relationship between these phenotypes and the phenotypes of *Microcystis* based on *16S, 16S-23S and* cpc*BA-IGS* because of the significant genetic variations among the strains of *Microcystis*.

### Polysaccharide biosynthesis-related genes

**Figure 6:**
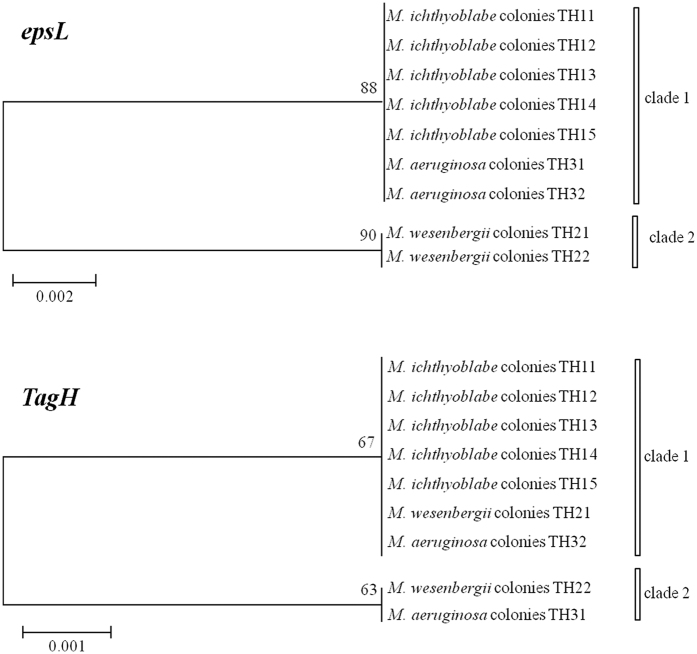
Phylogenetic tree based on the analysis of the polysaccharide biosynthesis-related gene sequences (*espL* and *TagH*).

Figure 6 shows a phylogenetic tree based on the analysis of the sequences of the polysaccharide biosynthesis-related genes (*espL* and *TagH*). The results demonstrate that the *M. wesenbergii* colonies could be divided directly from other colonies using *espL*. Xu *et al*.^27^ suggested that the polysaccharide biosynthesis-related gene *TagH* may explain the diversity of the *Microcystis* morphospecies. In the current study, *TagH* divided all of the samples into two clusters but failed to distinguish the different phenotypes.

**Figure 7:**
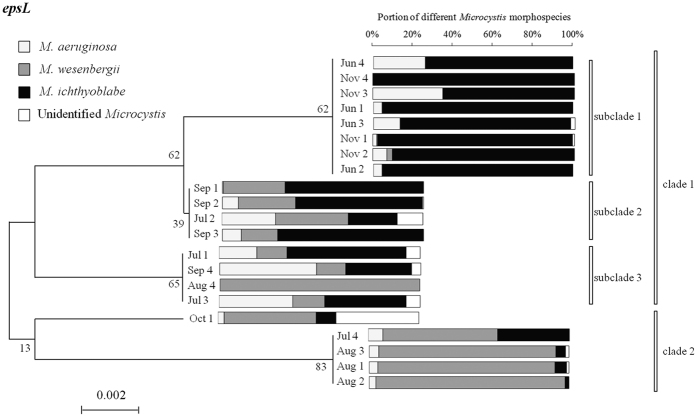
Phylogenetic tree based on the analysis of the sequences of *espL* genes extracted from *Microcystis* scums collected in different months with different morphospecies composition.

**Figure 8:**
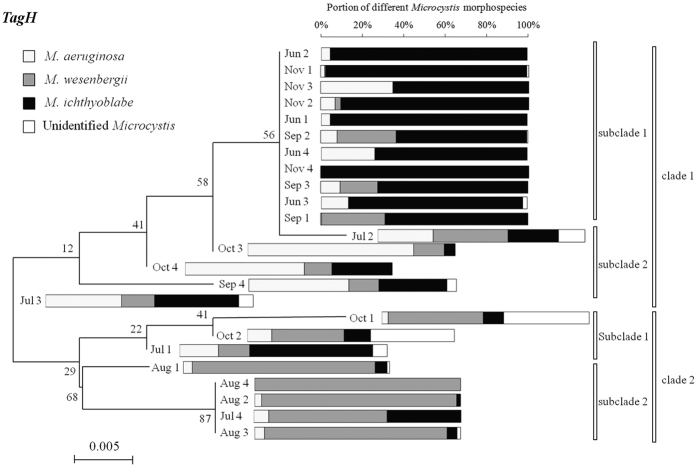
Phylogenetic tree based on the analysis of the sequences of *TagH* genes extracted from *Microcystis* scums collected in different months with different morphospecies composition.

Since very small amount of colonies were tested and cultured, there would be a risk that the final *Microcystis* morphotype would change compared with the initially identified *Microcystis* due to intraspecific competition. Therefore, part II was carried out to confirm as the polysaccharide biosynthesis-related genes could distinguish the *Microcystis* phenotypes. The phylogenetic tree based on the analysis of the sequences of the polysaccharide biosynthesis-related genes (*espL* and *TagH*) extracted from *Microcystis* “scum” collected from June and November 2015, was shown in Figs 7 and 8, respectively. The gene *espL* divided all of the samples into two clusters and the first cluster was divided into three subclades (Fig. 7). The samples in clade 2 was dominated by *M. wesenbergii* (60–100%). The samples in subclade 1 of clade 1 was dominated by *M. ichthyoblabe* (60–100%). As shown in Fig. 8, the gene *TagH* divided all of the samples into two clusters. All the samples collected in June and November were brought into subclade 1 in clade 1 and samples in August were brought into subclade 2 in clade 1. The former samples was dominated by *M. ichthyoblabe* (60–100%) and the latter samples was dominated by *M. wesenbergii* (60–100%). In consequence, it was confirmed that *M. wesenbergii* and *M. ichthyoblabe* could be distinguished by the polysaccharide biosynthesis-related genes *espL* and *TagH*. However, the two polysaccharide biosynthesis-related genes (epsL and TagH) may not be qualified for identifying all the species of *Microcystis*. These two genes combined with some other functional genes may succeed in identifying all the *Microcystis* species based on further researches.

Extracellular polysaccharide (EPS) was considered to be the material basis of *Microcystis* colony formation. A positive relationship between colony size and EPS content has been reported during recent years^51^,^35^. Li *et al*.^18^ illustrated that solubilization of mucilage, which consists of EPS, induced changes in *Microcystis* colonial morphology. Forni *et al*.^52^ indicated that the composition of EPS in different *Microcystis* species varied. The EPS content of various *Microcystis* morphospecies was also different^53^. Therefore, the content and composition of EPS has been postulated to be related to *Microcystis* colony morphology. In conclusion, the polysaccharide biosynthesis-related genes could distinguish the *Microcystis* phenotypes.

## Conclusions

1. *Microcystis* species identified by morphology or molecular tools (16S rDNA, 16S-23S ITS and *cpc*BA-IGS) could not be distinguished as toxic and non-toxic species.
2. There was no obvious relationship between the phenotypes of *Microcystis* species based on *16S, 16S-23S and* cpc*BA-IGS* because of the significant genetic variations among the strains of *Microcystis*.
3. It was confirmed that polysaccharide biosynthesis-related genes could distinguish the *Microcystis* phenotypes.

## Additional Information

**How to cite this article**: Xu, S. *et al*. Polysaccharide biosynthesis-related genes explain phenotype-genotype correlation of *Microcystis* colonies in Meiliang Bay of Lake Taihu, China. *Sci. Rep.* **6**, 35551; doi: 10.1038/srep35551 (2016).

## Figures and Tables

**Table 1:** List of primer pairs for the amplification and sequencing of *Microcystis.*

Primer	For sequence (5′–3′)	Rev sequence (5′–3′)	Reference
16S	ATGTGCCGCGAGGTGAAACCTAAT	TTACAATCCAAAGACCTTCCTCCC	Gan *et al*.^26^
ITS(A)	TCAGGTTGCTTAACGACCTA	(G/T)TTCGCTCGCC(A/G)CTAC	Otsuka *et al*. (1999a)
ITS(S)	CCAGTGAAGTCGTAACAAGG	GGGTT(T/G/C)CCCCATTCGG	Otsuka *et al*.(1999a)
*cpc*BA-IGS	GGCTGCTTGTTTACGCGACA	CCAGTACCACCAGCAACTAA	Otsuka *et al*. (1999b)
*mcy*B	CTATGTTATTTATACATCAGG	CTCAGCTTAACTTGATTATC	Neilan *et al*. (1995)
*eps*L	CGATGGGTGCGTTATCTTCC	GCCGATTACTGGCTGTCCTG	Gan *et al*.^26^
*Tag*H	CCGACAAAGGGACAGGTGAGA	CGCAAATCCTAAACGAGCCAC	Gan *et al*.^26^
